# Suprascapular Artery Variability in a Zambian Cadaveric Population: Anatomical Observations and Their Clinical Significance

**DOI:** 10.7759/cureus.99448

**Published:** 2025-12-17

**Authors:** Sunita Sethy, Vivienne Nambule Syamuleya, Amit Kumar Singh, Krupal J Joshi, Krishna Jasani

**Affiliations:** 1 Anatomy, Texila American University, Lusaka, ZMB; 2 Community and Family Medicine, All India Institute of Medical Sciences, Rajkot, IND

**Keywords:** shoulder anatomy, suprascapular artery, suprascapular nerve, suprascapular neuropathy, the cadaveric study

## Abstract

Introduction

The suprascapular artery typically arises from the thyrocervical trunk and traverses above the superior transverse scapular ligament (STSL). However, numerous variations in its origin and course have been documented, some of which may predispose individuals to suprascapular neuropathy or complicate surgical procedures of the neck and shoulder region. This study aimed to identify variations in the origin and course of the suprascapular artery in a Zambian cadaveric population.

Materials and methods

A descriptive cadaveric study was conducted over two years on 66 upper limbs from 33 embalmed cadavers of Zambian origin at Texila American University, Lusaka. Standard anatomical dissection techniques following Grant’s Dissector were used to expose the suprascapular artery, vein, and nerve. Ethical approval was obtained prior to conducting the study.

Results

Among the 66 upper limbs examined, one cadaver (1.5%) demonstrated an anomalous origin of the right suprascapular artery from the third part of the subclavian artery. In the same cadaver, both right and left suprascapular arteries exhibited an anomalous subligamentous course, passing beneath the STSL along with the suprascapular nerve (3%, 2/66). The left suprascapular artery, however, originated from the thyrocervical trunk and followed a normal proximal trajectory before deviating into an atypical subligamentous pathway at the suprascapular notch. In the remaining 32 cadavers, the suprascapular artery originated from the thyrocervical trunk and followed the typical suprascapular pathway above the STSL. No additional anatomical variations were observed in the suprascapular

Discussion

Although variations in the origin and course of the suprascapular artery have been previously reported, the coexistence of an anomalous origin with a bilateral subligamentous course is rare. Passage of the artery beneath the STSL may contribute to suprascapular nerve entrapment and can increase the risk of iatrogenic injury during neck dissections, clavicular surgeries, and shoulder procedures. Understanding such anatomical variations is crucial for clinicians managing unexplained shoulder pain, performing regional anesthesia, or operating in the cervicoscapular region.

Conclusion

This study identified a rare combination of variations involving anomalous origin and bilateral subligamentous course of the suprascapular artery in a Zambian cadaver from among 66 upper limbs examined from a total of 33 cadavers,. Although based on a limited sample, these findings emphasize the need for awareness of vascular variations in the suprascapular region to prevent diagnostic errors and surgical complications.

## Introduction

Anatomical variations of neurovascular structures in the cervicoscapular region hold significant clinical importance because they may influence the presentation of shoulder pain syndromes, contribute to suprascapular neuropathy, and increase the risk of iatrogenic injury during surgical or anesthetic procedures. Detailed knowledge of these variations is therefore essential for surgeons, orthopedists, radiologists, and anesthetists who routinely operate in or interpret imaging from the posterior cervical triangle and suprascapular region. Among the vessels of this region, the suprascapular artery (SSA) is particularly relevant due to its role in supplying the rotator cuff musculature and its close relationship with the suprascapular nerve.

The right subclavian artery arises from the brachiocephalic trunk, whereas the left subclavian artery originates directly from the aortic arch. Each artery is conventionally divided into three parts: the first part extends from its origin to the medial border of the scalenus anterior muscle; the second lies posterior to this muscle; and the third spans from the lateral border of the scalenus anterior to the outer margin of the first rib, where it continues as the axillary artery [1]. Branches of the first part include the vertebral artery, arising from its superoposterior aspect, and the internal thoracic artery arising inferiorly. The thyrocervical trunk, a short and wide vessel, typically originates from the anterior surface of the first part of the subclavian artery and rapidly divides into the inferior thyroid, suprascapular, and superficial cervical arteries [1].

The suprascapular artery usually represents the first (lower) branch of the thyrocervical trunk. It courses laterally through the posterior cervical triangle, positioned behind and superior to the clavicle [2]. During its course, it passes anterior to the scalenus anterior muscle, the phrenic nerve, the third part of the subclavian artery, and the primary cords of the brachial plexus [2]. It then proceeds posteriorly toward the scapula, crossing the superior border where it typically runs above the superior transverse scapular ligament (STSL) [2]. The accompanying suprascapular vein generally lies ventral and superior to the artery. After crossing the STSL, the artery enters the supraspinous fossa and continues to the infraspinous fossa through the spinoglenoid notch, participating in the scapular anastomotic network with branches of the dorsal scapular and circumflex scapular arteries [2].

Previous studies have documented considerable variation in the origin and course of the suprascapular artery. Natsis et al. identified five types of suprascapular notch morphology [3]. Anomalous origins have been reported from all three parts of the subclavian artery and the axillary artery, with a median incidence of approximately 10% [4]. Reported incidences range from 2.8% [5] to 21.3% [6]. Less commonly, the artery may arise from the internal thoracic artery (5.1%) [2,6,7], costocervical trunk (1%) [7], or dorsal scapular artery [8]. Rare cases of duplicated suprascapular arteries have also been described, with one branch following a normal suprascapular course and the anomalous branch passing beneath the STSL [9]. Complete absence of the artery has been reported in nearly 3% of cases [9].

Understanding these variations is critical, as the suprascapular artery supplies the rotator cuff musculature, and deviations in its anatomy may predispose patients to suprascapular neuropathy, contribute to unexplained shoulder pain, or complicate surgical interventions in the cervical and supraclavicular regions. Although numerous studies have reported variations in the suprascapular artery across different populations, data from sub-Saharan Africa remain extremely limited. Existing literature predominantly reflects findings from European, Asian, and North American cohorts, leaving a significant gap regarding whether these anatomical patterns apply to African populations, who may exhibit distinct morphologic characteristics. Addressing this lack of region-specific data is essential for improving the accuracy of clinical, surgical, and anesthetic procedures performed in African settings. This study aimed to document the origin and course of the suprascapular artery in a Zambian cadaveric sample, with emphasis on identifying clinically significant anatomical variations.

This study aimed to document the origin and course of the suprascapular artery in a Zambian cadaveric sample population, with emphasis on identifying clinically significant anatomical variations.

## Materials and methods

This cross-sectional cadaveric study was conducted over a period of two years in the Dissection Hall of Texila American University, Lusaka, Zambia. A total of 33 embalmed adult cadavers of Zambian (African) origin, yielding 66 upper limbs, were included. All cadavers were obtained through legally approved body donation procedures, and ethical clearance for the study was obtained from the Institutional Ethics Committee prior to commencement. Cadavers with evidence of prior neck, shoulder, or clavicular surgery, significant trauma, or malformations in the cervical or scapular regions were excluded to ensure accurate anatomical assessment. Each cadaver was assigned a unique identification code, and demographic details such as sex were recorded when available.

Standardized dissection protocols based on Grant’s Dissector were used to expose the suprascapular artery, suprascapular nerve, suprascapular vein, and adjacent structures. Dissection began with careful removal of the skin and superficial fascia in the posterior and anterior cervical triangles, followed by identification of key anatomical landmarks, including the clavicle, sternocleidomastoid muscle, trapezius muscle, and omohyoid muscle. The scalenus anterior muscle, phrenic nerve, and divisions of the brachial plexus were meticulously exposed to trace the origin and course of the suprascapular artery. Special care was taken to avoid disruption of natural anatomical relationships, particularly around the suprascapular notch and transverse scapular ligament. The origin, branching pattern, and course of the suprascapular artery were documented on each side, along with its relationship to the transverse scapular ligament. Variations were photographed using a high-resolution digital camera with appropriate labeling and scale markers. All observations were cross-verified by at least two anatomists to minimize observer error. The sample was treated as a random anatomical representation of the Zambian adult population, although without stratification by age or occupation due to limitations in cadaveric records. Both sides of each cadaver were examined independently to assess for bilateral symmetry or asymmetry in the origin and course of the suprascapular artery and its associated neurovascular structures

## Results

**Figure 1:**
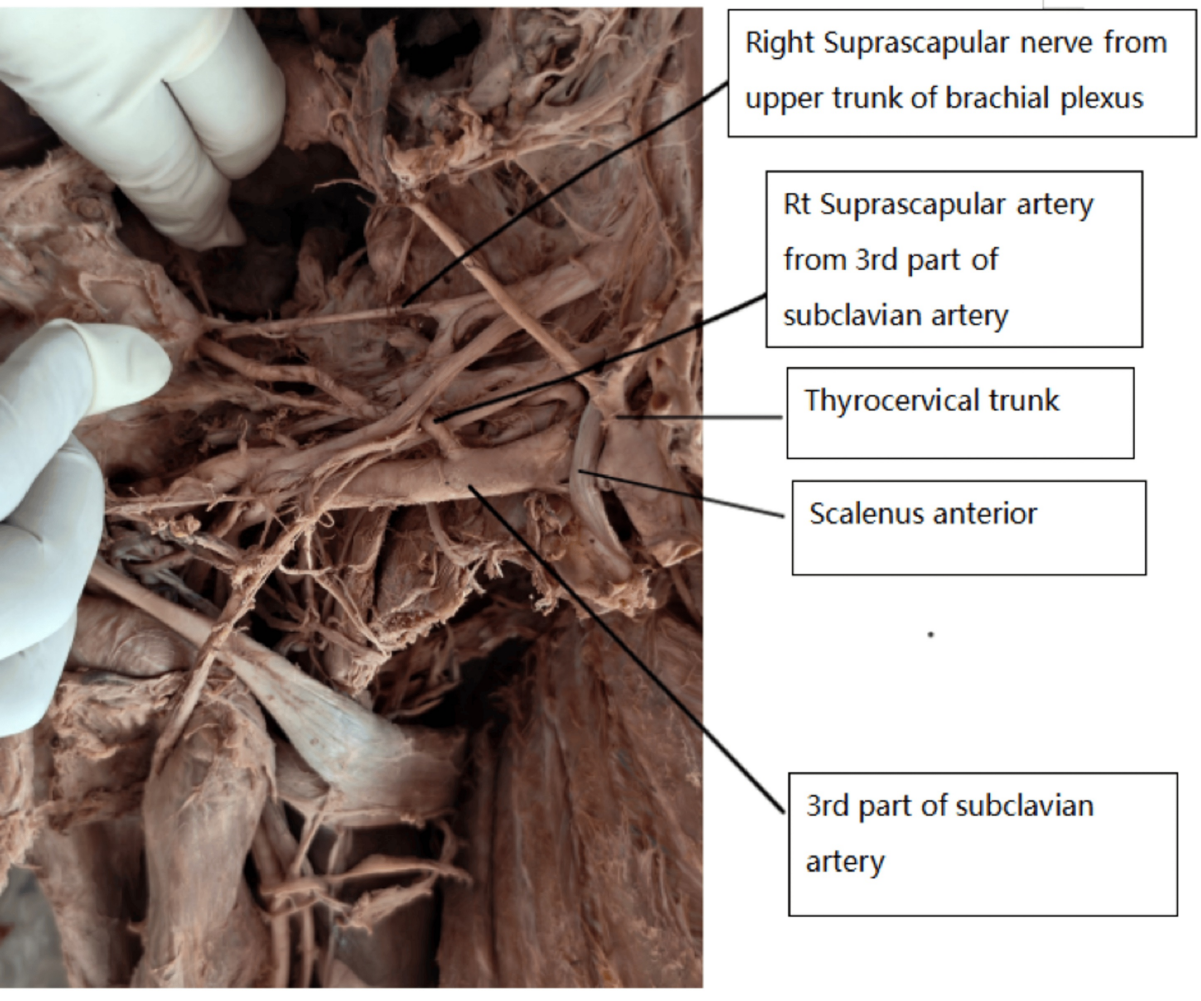
Right side of the neck. The image shows the subclavian artery, thyrocervical trunk, right suprascapular artery, right suprascapular nerve, upper trunk of the brachial plexus, and scalenus anterior muscle.

**Figure 2:**
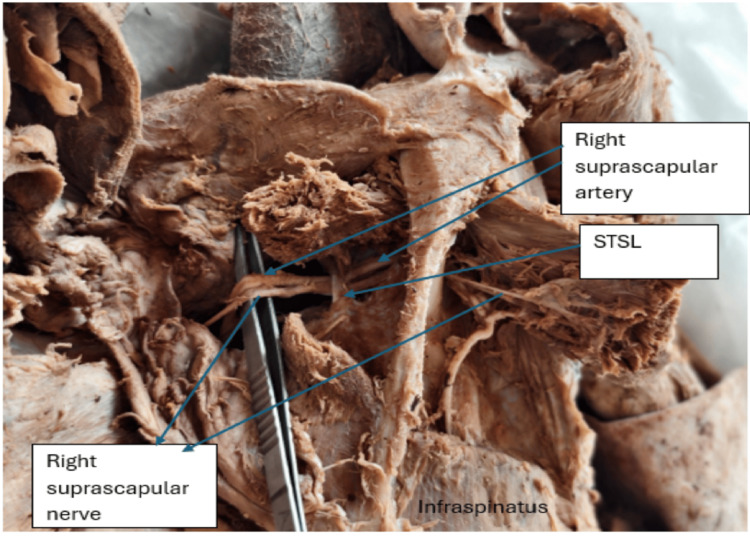
Right scapular region, posterior view. The image shows the right suprascapular artery and nerve passing under the superior transverse scapular ligament. STSL: superior transverse scapular ligament.

Of the 66 upper limbs examined, 64 were from male cadavers and two from a female cadaver. An anomalous origin of the suprascapular artery from the third part of the subclavian artery was observed on the right side in one cadaver, corresponding to an incidence of 1.5% (1/66). In this case, the artery arose lateral to the scalenus anterior muscle, passed between the divisions of the upper and middle trunks of the brachial plexus, and coursed posteriorly toward the suprascapular notch. Notably, both the suprascapular artery and suprascapular nerve passed beneath the STSL (Figures 1, 2).

**Figure 3:**
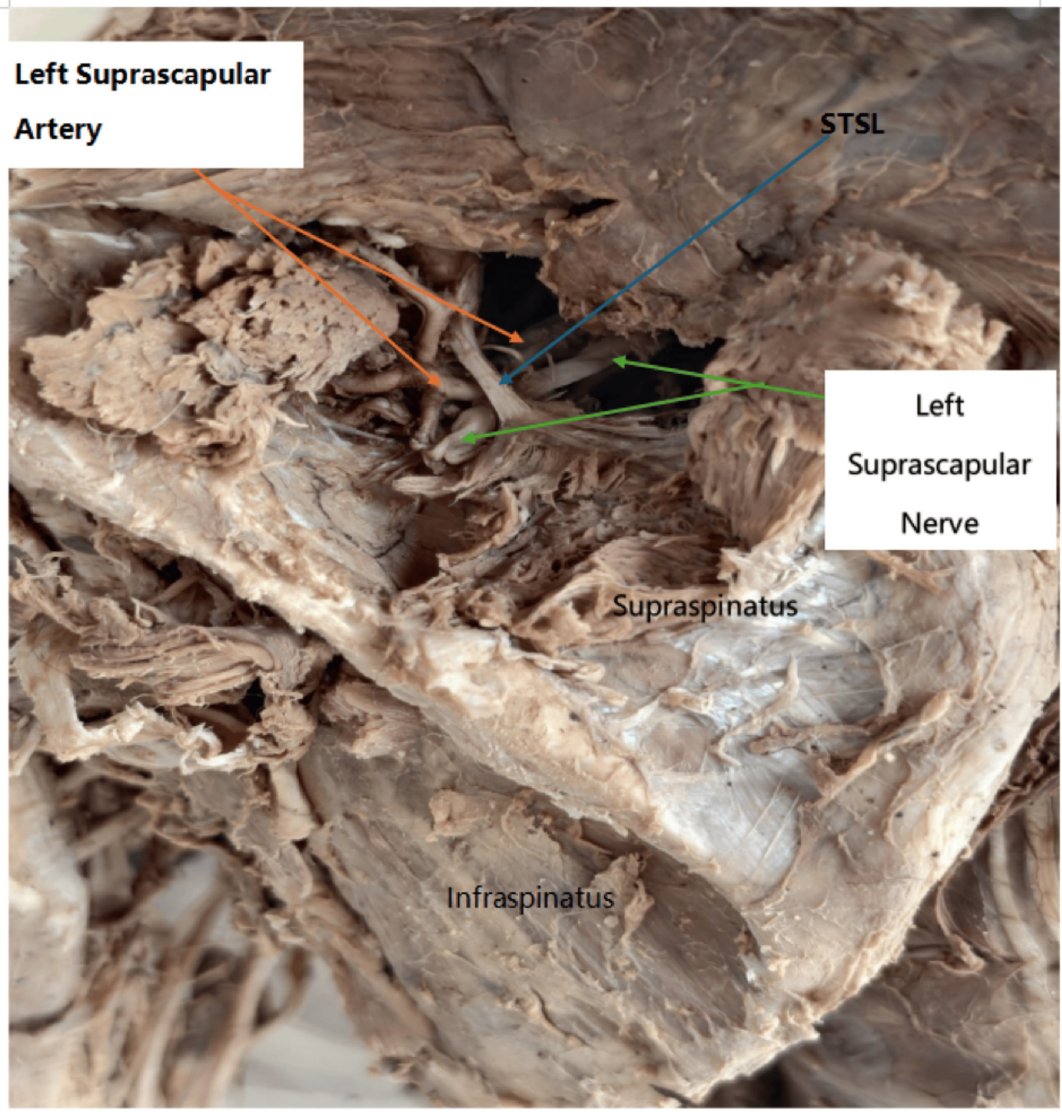
Left scapular region posterior view. The image shows the STSL, left suprascapular artery and left suprascapular nerve passing under the ligament. STSL: superior transverse scapular ligament.

The artery then entered the supraspinous fossa, supplying the supraspinatus muscle and contributing to scapular anastomosis. In this cadaver, the thyrocervical trunk on the right side gave rise to only two branches: the transverse cervical and the inferior thyroid arteries. The costocervical trunk originated from the second part of the subclavian artery, and the dorsal scapular artery arose from the third part. Upon dissection of the contralateral (left) upper limb of the same cadaver, the suprascapular artery originated from the thyrocervical trunk but followed an anomalous subligamentous course. The artery, vein, and nerve all passed beneath the STSL (Figure 3). The suprascapular veins were sectioned during dissection to allow clear visualization of the artery. In the remaining 32 cadavers (64 upper limbs), the suprascapular artery originated from the thyrocervical trunk and followed the typical course above the STSL.

## Discussion

In the present study of 66 upper limbs from 33 cadavers of Zambian origin, we observed a rare combination: on one side (right), the suprascapular artery (SSA) originated from the third part of the subclavian artery, lateral to the scalenus anterior muscle and then coursed beneath the superior transverse scapular ligament (STSL) along with the suprascapular nerve and vein. On the contralateral (left) side of the same cadaver, SSA had the typical origin (from the thyrocervical trunk), but its course was anomalous: the artery, vein, and nerve again passed beneath the STSL. This bilateral subligamentous course of the SSA/vein/nerve in the same individual, combined with anomalous origin on one side, represents a distinct anatomical variation that may carry important clinical implications.

The suprascapular artery plays a central role in supplying the rotator cuff musculature [10]; therefore, variations in its origin or course have important clinical and surgical implications. When the artery passes beneath the STSL, the suprascapular notch becomes increasingly crowded, predisposing the suprascapular nerve to compression. This may result in suprascapular neuropathy, characterized by chronic shoulder pain, weakness of abduction and external rotation, and atrophy of the supraspinatus and infraspinatus muscles [11,12].

Additional factors, such as ossification of the STSL or an enlarged suprascapular artery, may further narrow the notch and exacerbate nerve entrapment [11,13]. Vascular variations may also contribute to neuropathy via ischemic mechanisms; microemboli in the vasa nervorum of the suprascapular nerve have been reported after injury to the suprascapular artery [14].

From a diagnostic standpoint, patients presenting with refractory, non-arthritic shoulder pain or rotator cuff symptoms may actually have neuropathy related to vascular or morphological variations at the notch. Awareness of such anatomical patterns is crucial for accurate diagnosis, planning of surgical decompression, regional anesthesia, and avoiding iatrogenic injury during neck dissections or supraclavicular procedures.

Comparison with previous literature on origin variations

Classical anatomical references report that the SSA most commonly originates from the thyrocervical trunk of the subclavian artery [15]. The incidence of SSA arising from other sources, such as the internal thoracic artery, the dorsal scapular artery, the costocervical trunk, or directly from the subclavian or axillary arteries, is low [15]. Specifically, a study by Huston L et al. estimated that SSA may arise from subclavian or axillary arteries in up to ~10% of cases [16]. Another cadaveric series documented SSA arising from the third segment of the subclavian artery in 1.6% of specimens (1/62 sides), a similar frequency to our observation (1.5%, 1/66) [11]. Our finding thus aligns with this rare but documented variation. The occurrence in a Zambian population suggests that such anatomical variants are not confined to any single ethnic or racial group. Additionally, the fact that only one limb (right) in our sample showed anomalous origin underscores the low but real prevalence of such variants, consistent with previously published data.

Comparison with previous literature on the course, subligamentous passage

Most classical and cadaveric studies describe the SSA, along with the suprascapular vein (SSV) and nerve (SSN), as passing over the STSL at the suprascapular notch [17]. However, substantial anatomical variability has been documented. In a large cadaveric series of 106 shoulders, authors categorized four types of neurovascular arrangement relative to STSL: Type I (~61.3%): artery above ligament, vein and nerve below, Type II (~17%): both vessels above, nerve below, Type III (~12.3%): artery, vein, and nerve all below the ligament, Type IV (~9.4%): other variants [14].

Type III (all structures under STSL) is considered anatomically significant as it reduces the suprascapular opening and may predispose to nerve entrapment [14]. In the classical series by Tubbs RJ et al., subligamentous passage of SSA with SSN was reported in ~2.5% of 120 cadavers, including a bilateral case [18]. Our present case resembles those rare Type III or bilateral subligamentous patterns, but with additional complexity: one side (right) showed an anomalous origin from the third part of the subclavian artery, while the contralateral side had the normal origin but still followed an atypical subligamentous course. To our knowledge, such a combination of bilateral subligamentous passage in the same individual with asymmetrical origin variation is infrequently reported, if at all. Most prior reports describe either origin variation without bilateral subligamentous course, or subligamentous course with typical origin [18]. Furthermore, in a recent case reported in 2023, an aberrant SSA originating from the axillary artery was observed bilaterally along with other neurovascular variants; in that case, on at least one side, the SSA coursed beneath the STSL [19]. This supports the notion that such combined variations are possible, albeit rare.

Clinical and surgical implications, critical reflection

Given that the Type III configuration (artery, vein, and nerve beneath STSL) reduces the area of the suprascapular notch, as documented by morphometric studies, such anatomy may predispose to nerve entrapment and suprascapular neuropathy [14]. In our cadaver, the bilateral subligamentous course, in the presence of an anomalous origin on one side, could represent a potentially higher-risk anatomical configuration. Moreover, aberrant origin from the third part of the subclavian artery may place the SSA in an unusual course relative to the brachial plexus or clavicle, potentially increasing the risk of iatrogenic injury during neck dissections, clavicular surgeries, or regional anesthesia. Previous authors have emphasized the surgical relevance of such variants, especially when planning decompression at the suprascapular notch, vascular reconstructions, or flap harvests [19]. Our finding highlights that even in individuals with a normal SSA origin (thyrocervical trunk), subligamentous passage may occur, suggesting that origin alone cannot reliably predict the course. Therefore, preoperative imaging or careful dissection becomes essential.

The present study has several limitations that should be considered when interpreting the findings. First, the sample size was relatively small, comprising 33 cadavers (66 upper limbs), which may limit the generalizability of the observed anatomical variations to the wider Zambian or sub-Saharan African population. Second, detailed morphometric measurements of the suprascapular notch, superior transverse scapular ligament, and neurovascular bundle were not performed, restricting our ability to quantify the degree of potential compression or correlate it with established anatomical risk factors for suprascapular neuropathy. Third, the cadavers' clinical histories were unavailable, preventing any assessment of whether the identified variations were associated with symptoms in life. Lastly, as this was a single-centre descriptive cadaveric study, population-level prevalence and ethnic variation could not be conclusively established; multicentric studies with larger and more diverse samples are needed to validate and expand upon these findings. The study could not be stratified by variables such as age or occupation because these details were not available in the cadaveric records, which may have limited further correlation with demographic or occupational risk factors

## Conclusions

This study documents a rare combination of variations in the origin and course of the suprascapular artery within the Zambian cadaveric samples, including an anomalous origin from the third part of the subclavian artery and bilateral subligamentous passage beneath the superior transverse scapular ligament. Although based on a modest sample size, these findings add region-specific anatomical data to the existing literature and highlight the spectrum of suprascapular vascular variations that may occur in African populations. The observed patterns have important clinical relevance, as deviations in the artery’s origin or course may contribute to suprascapular nerve compression or increase the risk of iatrogenic injury during neck and shoulder procedures. Greater awareness of these variations is essential for surgeons, anesthesiologists, and clinicians involved in the evaluation and management of shoulder pain, regional anesthesia, and operative planning in the cervicoscapular region.
